# Risk factors associated with continuous ambulatory peritoneal dialysis-related infections in chronic kidney disease patients at Dr. Kariadi Hospital Semarang

**DOI:** 10.1017/ash.2025.131

**Published:** 2025-09-03

**Authors:** Paramestri Sekar Kinanthi, Ayudyah Nurani, Retty Kharisma Sari, Nur Farhanah, Muchlis Achsan Udji Sofro

**Affiliations:** 1 Internal Medicine Resident, Department of Internal Medicine, Faculty of MedicineDiponegoro University; 2 Nephrology and Hypertension Division, Department of Internal Medicine, Faculty of MedicineDiponegoro University; 3 Tropical Medicine and Infectious Disease Division, Department of Internal Medicine, Faculty of MedicineDiponegoro University

**Keywords:** Continuous Ambulatory Peritoneal Dialysis-Related Infections, Risk factors

## Abstract

**Introduction:** Continuous Ambulatory Peritoneal Dialysis (CAPD) is a treatment method for Chronic Kidney Disease (CKD) that allows patients to undergo dialysis therapy at home. Although CAPD provides benefits in terms of flexibility, efficiency, and comfort, patients undergoing CAPD are at high risk of infections, including exit site infections, tunnel catheter infections, and Peritoneal Dialysis (PD) peritonitis. This study aims to identify risk factors associated with CAPD infections in CKD patients at Dr. Kariadi Hospital, Semarang, Indonesia. **Methods:** A retrospective cross-sectional study design was applied to adult CKD patients undergoing CAPD at Dr. Kariadi Hospital between January 2022 and March 2024. Data were collected from patients’ medical histories and records, then analyzed using SPSS 21. A p-value less than 0.05 was used to determine statistically significant variables. **Results:** This study involved 81 adult patients undergoing CAPD with 58% male subjects. There were 23 (31.9%) subjects who experienced CAPD infections. Subjects who had infections experienced exit-site infections (10,5%) and peritonitis (89,5%). The most dominant microorganism in infected patients was Staphylococcus epidermidis. Diabetes mellitus (p = 0.03) contributed as significant risk factors for infection, while hypoalbuminemia and overweight were not significant risk factors (p > 0.05). **Conclusion:** In conclusion, the incidence of CAPD-related infections was high with a predominance of Staphylococcus epidermidis. Diabetes mellitus is considered a contributing factor to the infection.

